# Supplementary material for: Transformation of the head-direction signal into a spatial code
Source: Nat Commun. 2017 Nov 24;8:1752. doi: 10.1038/s41467-017-01908-3 (PMC5700966; doi:10.1038/s41467-017-01908-3)
Supplement: Supplementary file 1 — Supplementary Information [file 41467_2017_1908_MOESM1_ESM.pdf]

# Transformation of the head-direction signal into a spatial code

Adrien Peyrache[1,3], Natalie Schieferstein[1,†], Gyorgy Buzsaki[1,2]

[1] The Neuroscience Institute, Center for Neuroscience, [2] New York University Langone Medical Center, New York City, New York, USA

[3] Department of Neurology and Neurosurgery, Montreal Neurological Institute, McGill University, Montreal, Quebec, Canada

[†] Present address: Institute for Theoretical Biology, Department of Biology, Humboldt-Universität zu Berlin, 10115 Berlin, Germany

Correspondence should be addressed to A. P. (adrien.peyrache@mcgill.ca) and G. B. (gyorgy.buzsaki@nyumc.org)

**Supplementary Materials:**

Supplementary Figures 1–6

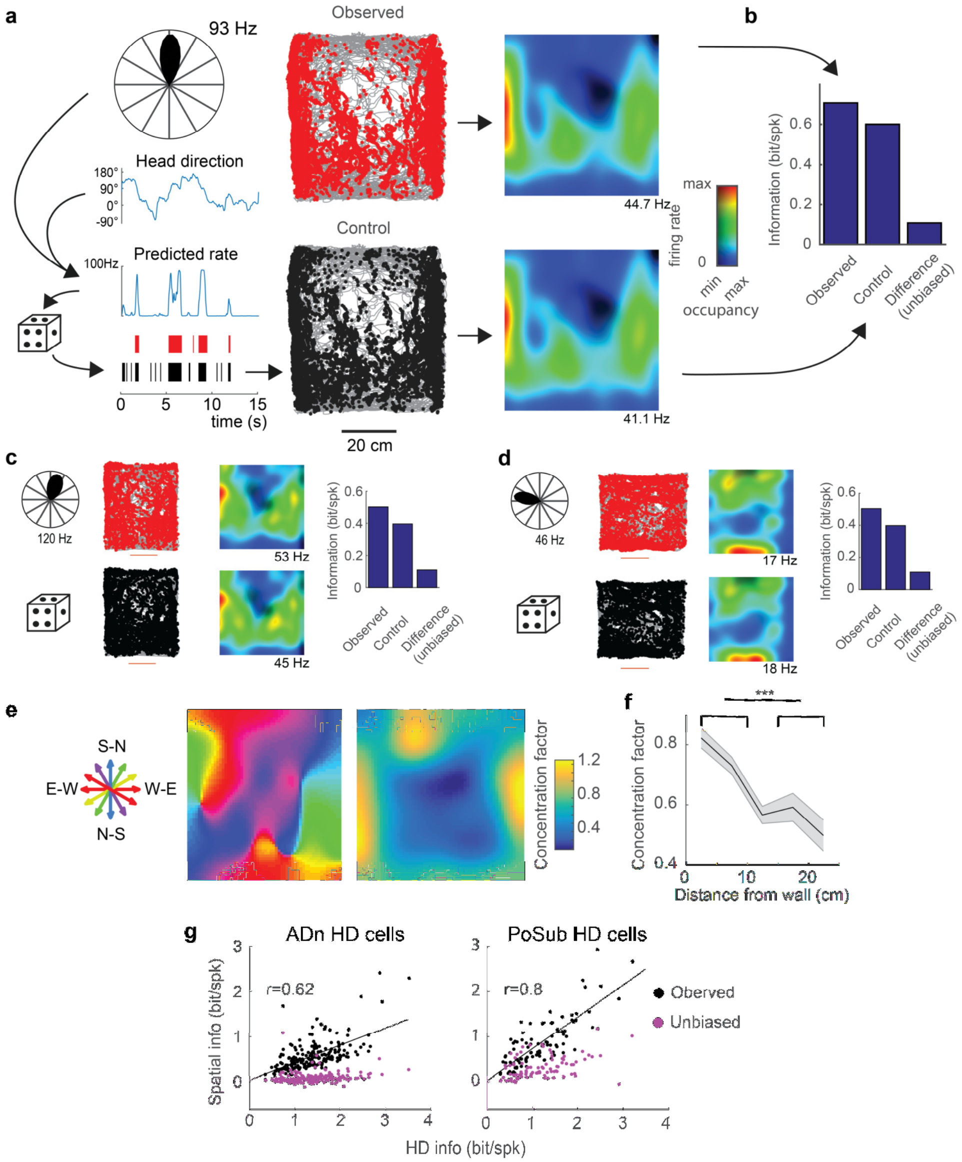


**Supplementary Fig. 1** HD cells convey spatial information. **a** Example of a HD cells recorded in the ADn. *Top left*, HD tuning curve (average firing rate in function of animal's orientation); *top middle*: position of the animal (*grey*) superimposed with location of the animal when the neuron spiked (*red dots*); *top right*, spatial tuning of the neuron where average firing rate at each location is represented as a colormap. Contrast displays the overall occupancy at each location. *Bottom*, same as top panels for a random spike train generated from the HD tuning curve only. **b** Spatial information conveyed by the neuron and by the randomly generated spike train. The difference between these two measures is referred to as 'unbiased information'. **c–d** Two other examples of HD neurons recorded from the ADn presented as in **a–b**. **e** *Left*, average orientation of animal's displacement for the session shown in (A); *right*; concentration factor of these orientations around their mean. North ("N") and South ("S") orientations occurred more frequently along the East ("E") and West ("W") walls because mice rarely run straight all the way to the walls. Instead, they turn and run parallel with the wall. Similarly, E and W orientations concentrated more frequently along the N and S walls. The animal's orientation bias was measured as the concentration of the animal's heading in the two possible directions parallel to the walls.
**f** Average (± s.e.m.) concentration of orientation as a function of distance to the nearest wall. Concentration was significantly higher 10 cm or less from the nearest walls than at the center of environment, measured between 15-25 cm from the walls ($p<10^{-7}$, Mann-Whitney U test). **g** Spatial and HD information rates were positively correlated in both ADn (black dots in Figure 3A, b; r = 0.59, n=205, $p<10^{-10}$, Pearson's test) and PoSub (r = 0.81, n=88, $p<10^{-10}$, Pearson's test). This correlation did not depend on other factors, such as firing rate difference between neurons (partial correlations with firing rates accounted for less than 5% of the HD-spatial information correlation).

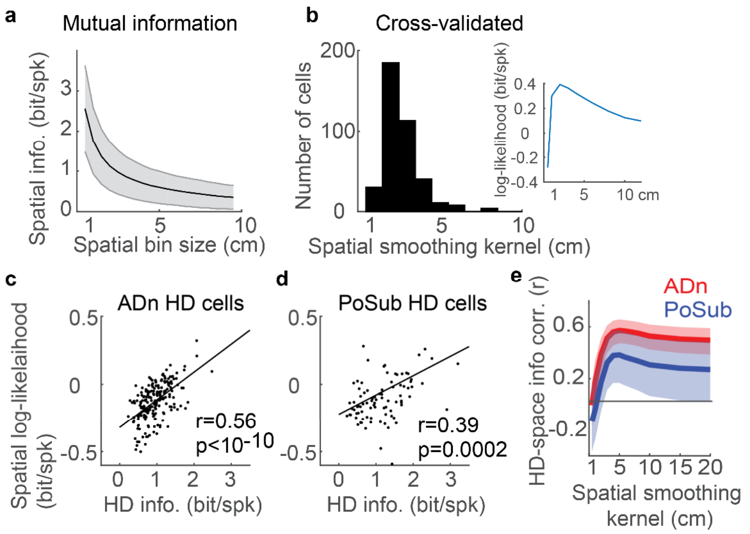


**Supplementary Fig. 2** Cross-validated spatial information measures confirm the HD-spatial information relationship. **a** Mutual information decreases with the size of spatial bins (mean ± s.d.). **b** Distribution of spatial kernel widths that maximized the cross-validated spatial log-likelihood; inset displays the example of spatial log-likelihood in function of spatial smoothing width for an example neuron. **c** Spatial log-likelihood versus HD information for ADn HD neurons. **d** Same as **c** for HD neurons recorded in the PoSub. **e** Correlation between spatial log-likelihood and HD information versus spatial smoothing width for ADn (*red*) and PoSub (*blue*) HD neurons; shaded areas represent 95% interval of confidence. The correlation showed a maximum for a spatial binning of 4–6cm.

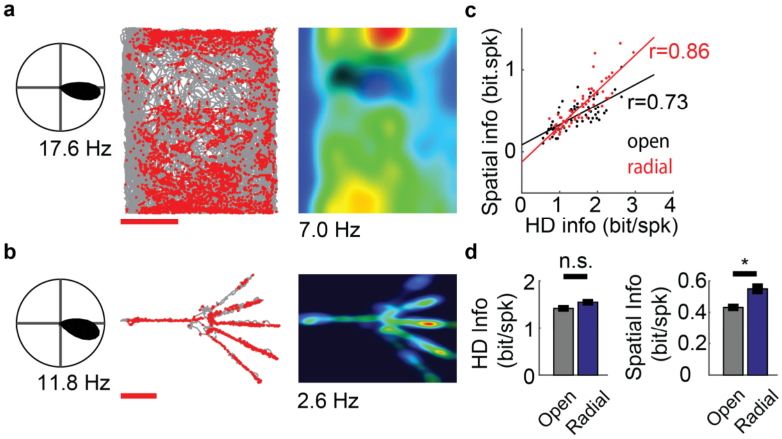


**Supplementary Fig. 3** HD and spatial information correlation is modulated by the environmental constrain on animal's behavior. **a** Example of a HD cell recorded in the ADn in an open environment, presented as in Figure 1 and Supplementary Fig. 1. **b** The same neuron now recorded in a 6 arm maze. **c** Neuron-by-neuron correlation of HD and spatial information and linear of best linear fit ($p<10^{-10}$ for both conditions, n=57, Pearson's test). The 'gain' (slope of spatial versus HD information) was stronger in the radial arm maze than in the open field ($p=7.7\ 10^{-5}$, t-test). **d** Spatial, but not HD, information was increased in the 6-arm maze. Average (± s.e.m.) HD (*left*) and spatial (*right*) information in both environments (*p<0.01, sign test; n.s. p>0.05)

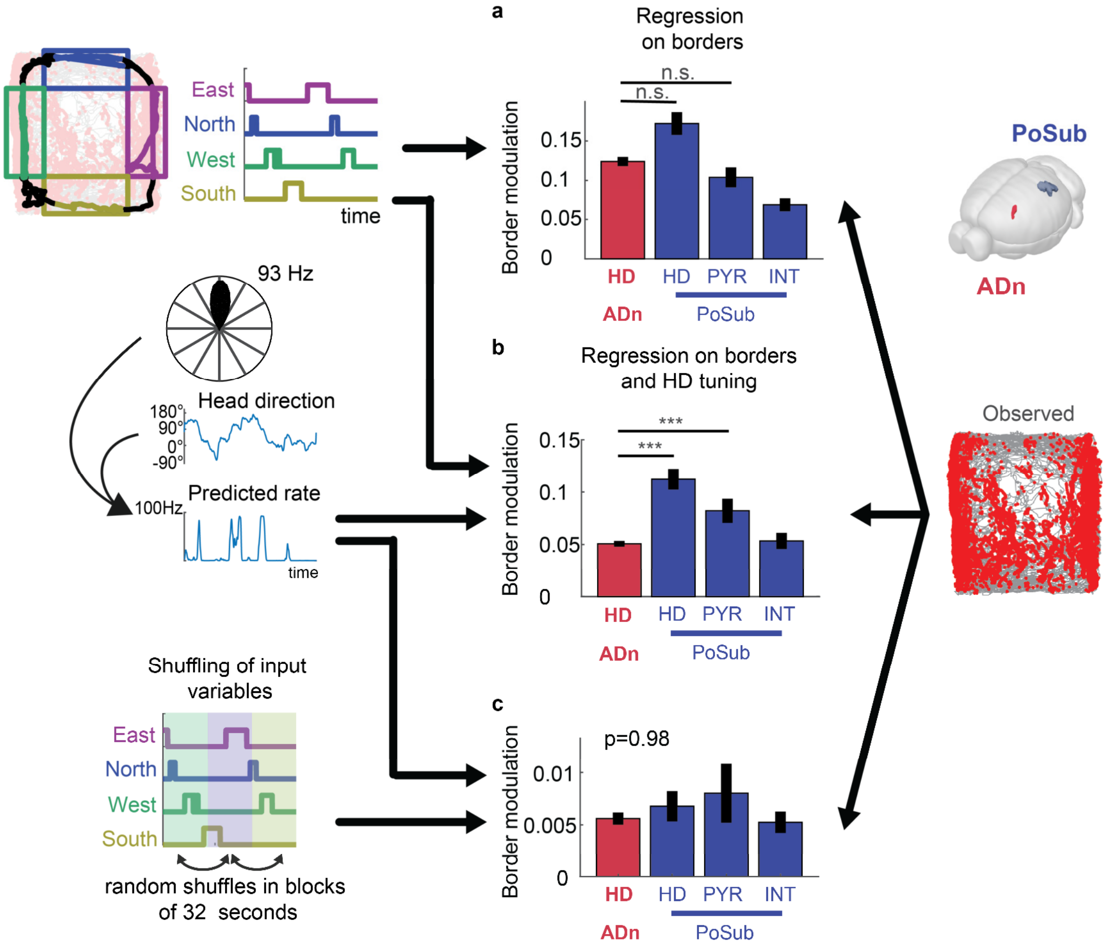


**Supplementary Fig. 4** Regression of neuronal data with Generalized Linear Model. **a** Binned spike trains were regressed on binary variables indicating the presence of the animals along each of the wall (as shown for an example trajectory on the *left*) using a log link function, assuming neurons are Poisson processes. Bar plot displays average (± s.e.m.) maximal regression coefficients (among the four coefficients associated with each wall, referred to as 'border modulation') for each group of neurons. The modulation of HD neurons by borders were not different in the ADn and the PoSub. 'HD': HD neurons; 'PYR': putative pyramidal cells; 'INT': putative inhibitory interneurons. The distinction between pyramidal cells and interneurons was assessed by difference in waveform features (see Methods). **b** The same regression was then computed by adding the expected firing rate based on animal's behavior and HD tuning curves (as shown on the *left* panel and in Supplementary Fig. 1). The modulation of ADn HD neurons by border is now dramatically reduced, indicating that this modulation was mainly explained by the inhomogeneous direction-by-position distribution of the animal in its environment. This was not the case however of PoSub HD and non-HD pyramidal neurons, suggesting that these neurons convey an additional information related to borders. **c** Any signal can always be fitted to random variables. To test whether the higher modulation by borders in the PoSub was not a by-product of non-specific regression against unexplained variance, variables coding for the borders were randomly shuffled in time, in blocks of 32 seconds to preserve the global statistics of the inputs. There was no difference between the resulting modulation coefficients (p=0.98, ANOVA). Note the change in the scale of the y-axis. Brain illustration: © 2015 Allen Institute for Brain Science. Brain Explorer. http://mouse.brain-map.org/static/brainexplorer

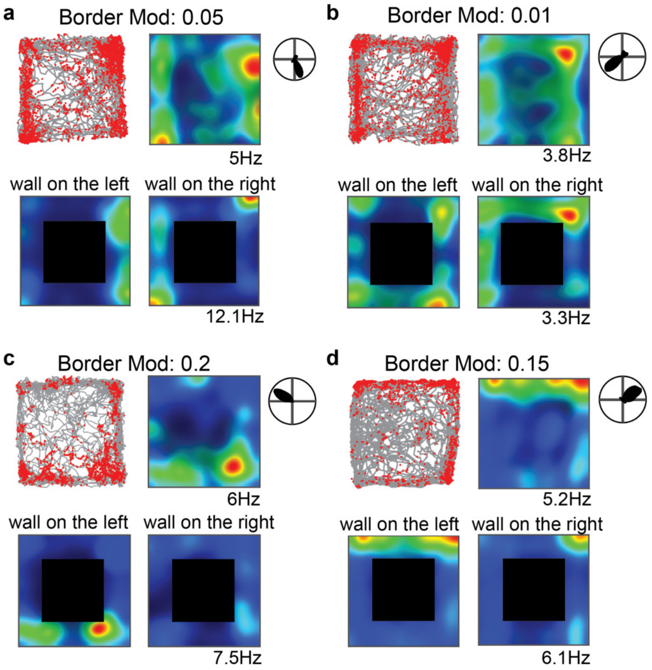


**Supplementary Fig. 5** Diversity of HD and border modulation in the PoSub. **a–d** Additional examples of PoSub HD cells. *Top* panels, from *left* to *right*: animal's trajectory (*grey*) and spike location (*red dots*), place fields and HD tuning curve. Bottom panels display place fields restricted to the 'wall on the left' (*left*) and 'wall on the right' (*right*) conditions. **a,b** Two neurons with very low border modulation (example displayed in **b** is from the same session as in Fig. 1**a–d**). **c,d** Two neurons modulated by body-wall relationship.

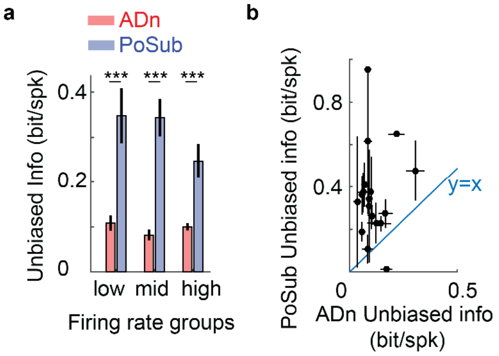


**Supplementary Fig. 6** Unbiased information difference between ADn and PoSub HD neurons did not result from non-specific parameters. **a** Average (± s.e.m.) unbiased spatial information in the ADn and the PoSub as a function of average firing rate (low: $<$ 1 Hz; mid: 1Hz – 2 Hz; high: $\geq$ 2 Hz). Increase in unbiased spatial information did not depend on firing rates ($p>0.05$; two-way ANOVA; N=31, 26, 31 PoSub neurons and N=31, 29, 117 ADn neurons in each group); *** $p<0.001$ (Mann-Whitney U test).

**b** Average unbiased spatial information was significantly higher in the PoSub than in the ADn in 18 (out of 19) sessions (three animals) where the two neuronal populations were simultaneously recorded ($p<0.001$; Mann-Whitney U test).
